# Dysfunction of von-Hippel Lindau factor causes reduced degradation of HIF leading to renal cancer. Hypoxia-inducible factor-prolyl hydroxylase enzyme inhibitors also lessen HIF destruction and could therefore increase renal cancer

**DOI:** 10.3389/fphar.2023.1170796

**Published:** 2023-06-06

**Authors:** Helge Waldum

**Affiliations:** Department of Clinical and Molecular Medicine, Faculty of Medicine and Health Sciences, Norwegian University of Science and Technology, Trondheim, Norway

**Keywords:** von Hippel Lindau factor, hypoxia inducible factor (HIF), renal cancer, prolyl hydroxylase inhibitor (roxadustat), renal anemia, erythropoietin producing cell

## HIF and renal cancer

Treatment of anemia due to chronic kidney disease with agents increasing erythropoietin (EPO) from erythropoietin producing cell (EPC) by inhibition of one of the enzymes causing degradation of hypoxia inducible factor (HIF) (HIF prolyl hydroxylase inhibitor (HIF-PHI)) is gaining popularity (Chen et al., 2022). It is, however, well-known that a small proportion of patients with clear cell renal cell cancer (CCRCC) has erythrocytosis and a larger part EPO production in the cancer cells (Palapattu et al., 2002; Wiesener et al., 2007). In a study applying immunohistochemistry with high sensitivity we found that practically all CCRCCs expressed erythropoietin as well as the neuroendocrine marker neuron-specific enolase (NSE) (Mjønes et al., 2017), the latter a marker with hitherto misrecognized reputation.We concluded that CCRCC (the dominating histological type of kidney cancer) (Mjønes et al., 2017; Waldum et al., 2022) originated from the EPC. There are many features in CCRCC in common with neuroendocrine tumours (Waldum et al., 2022). HIF is a major stimulator of EPO production, whereas von-Hippel Lindau factor is central in HIF degradation. Dysfunction of von-Hippel Lindau factor is an important factor in CCRCC demonstrating the role of HIF in renal carcinogenesis (Gnarra et al., 1994). There is generally a close correlation between stimulation of the function and proliferation of a cell. Chronic overstimulation of the cell of origin (whether a tubular cell or based upon our study, the EPO cell) by HIF predisposes to renal cancer in von-Hippel Lindau syndrome (Ohh et al., 2022). and it is reason to fear that increased stimulation of the EPO cell by elevated HIF due to inhibited degradation by HIF-PHI inhibitors including roxadustat, could in long-term predispose to CCRCC. The importance of long-term hyperstimulation by HIF in renal carcinogenesis is demonstrated by the latency of renal cancer even in patients with congenital von-Hippel Lindau syndrome. However, there is no study describing any neoplastic renal changes in human secondary to roxadustat although growth of renal cysts has been reported (Zhu et al., 2022). Lack of neoplastic renal changes in long-term rodent studies are also reassuring although the dosing of roxadustat was difficult due to rapid clearance. Although toxicology studies hitherto seem reassuring, taking into consideration the long latency for neoplasia in general, there is every reason to be vigilante concerning long-term use of agents inducing increased HIF exposure.

## References

[B1] Chen T. Huang J. Dong H. Xu L. Chen C. Tang Y. (2022). Efficacy and safety of roxadustat for the treatment of anemia in non-dialysis chronic kidney disease patients: A systematic review and meta-analysis of randomized double-blind controlled clinical trials. Front. Nutr. 9, 1029432. 10.3389/fnut.2022.1029432 36466382 PMC9710737

[B2] Gnarra J. R. Tory K. Weng Y. Schmidt L. Wei M. H. Li H. (1994). Mutations of the VHL tumour suppressor gene in renal carcinoma. Nat. Genet. 7, 85–90. 10.1038/ng0594-85 7915601

[B3] Mjønes P. G. Nordrum I. S. Qvigstad G. Sørdal Ø. Rian L. L. Waldum H. L. (2017). Expression of erythropoietin and neuroendocrine markers in clear cell renal cell carcinoma. Apmis 125, 213–222. 10.1111/apm.12654 28233444

[B4] Ohh M. Taber C. C. Ferens F. G. Tarade D. (2022). Hypoxia-inducible factor underlies von Hippel-Lindau disease stigmata. Elife 11. 10.7554/eLife.80774 PMC9427099 36040300

[B5] Palapattu G. S. Kristo B. Rajfer J. (2002). Paraneoplastic syndromes in urologic malignancy: The many faces of renal cell carcinoma. Rev. Urol. 4, 163–170. 16985675 PMC1475999

[B6] Waldum H. Mjønes P. (2022). “Clear cell renal cancer, a tumour with neuroendocrine features originating from the erythropoietin-producing cell,” in Renal cell carcinoma - recent advances, new perspectives and applications [working title]. Editor Chen J. (London, England: IntechOpen).

[B7] Wiesener M. S. Münchenhagen P. Gläser M. Sobottka B. A. Knaup K. X. Jozefowski K. (2007). Erythropoietin gene expression in renal carcinoma is considerably more frequent than paraneoplastic polycythemia. Int. J. Cancer 121, 2434–2442. 10.1002/ijc.22961 17640059

[B8] Zhu X. Jiang L. Wei X. Long M. Du Y. (2022). Roxadustat: Not just for anemia. Front. Pharmacol. 13, 971795. 10.3389/fphar.2022.971795 36105189 PMC9465375

